# Prognostic performance of examined lymph nodes, lymph node ratio, and positive lymph nodes in gastric cancer: a competing risk model study

**DOI:** 10.3389/fendo.2025.1434999

**Published:** 2025-02-21

**Authors:** Xiao Gu, Yaqi Du

**Affiliations:** ^1^ Department of Oncology, Shengjing Hospital of China Medical University, Shenyang, China; ^2^ Department of Gastroenterology, The First Hospital of China Medical University, Shenyang, China

**Keywords:** gastric cancer, competing risk model, lymph node ratio, examined lymph node, positive lymph node, prognosis

## Abstract

**Background:**

Previous research on the prognostic effectiveness of examined lymph nodes (ELN), lymph node ratio (LNR), and positive lymph nodes (pN) in postoperative gastric cancer (GC) has yielded inconsistent results despite their widespread use.

**Methods:**

This study used a competing risk model (CRM) to evaluate the prognostic efficacy of these markers in patients with GC. Data from 337 patients with lymph node (LN)-positive stage II GC undergoing resection and chemotherapy between 2010 and 2015 were collected from the Surveillance, Epidemiology, and End Results database. Optimal cutoff values for ELN and LNR were determined using restricted cubic splines, and pN was divided into three groups based on the AJCC staging system. The survival analyses were conducted using Kaplan–Meier curves, Cox proportional hazards analysis, cumulative incidence curves, and CRM. Subgroup analysis and interaction tests were performed to evaluate the correlation between LN status and survival within subgroups.

**Results:**

The results indicated that the optimal cutoff values for ELN, LNR, and pN were 16, 0.1, and 2. Multivariate Cox analysis showed that ELN (hazard ratio [HR] = 0.67), LNR (HR = 2.23), and pN (HR = 2.80) were independent predictors of overall survival, whereas only LNR (HR = 2.08) was independently associated with disease-specific survival. The CRM revealed that LNR (sub-distribution hazard ratio [SHR] = 1.89) and pN (SHR = 2.80) were independently associated with disease-specific survival.

**Conclusion:**

In conclusion, ELN, LNR, and pN are all significant predictors of overall survival for GC. However, LNR demonstrates stronger robustness in predicting DSS than ELN and pN. The LNR may supplement the TNM staging system in identifying prognostic discrepancies.

## 1 Introduction

Gastric cancer (GC) is the fifth most diagnosed cancer and the fourth leading cause of cancer-related mortality worldwide. In 2020, 1,089,103 new cases of GC were reported, with 768,793 deaths (1). GC can be classified into four major types: adenocarcinoma, adenosquamous carcinoma, squamous carcinoma, and carcinoid. Adenocarcinoma is the most common histopathological type and represents an overwhelming majority (> 90%) of GC cases (2). The prognosis of patients with GC has improved owing to recent advances in comprehensive treatment. In a phase III randomized trial, patients with stage II GC who received adjuvant chemotherapy had a 5-year overall survival (OS) rate of 84.2% (3). LN status is a strong prognostic factor in patients with GC. The 5-year OS rate decreased to 30% once lymph node (LN) metastasis occurred (4).

Currently, the number of positive LNs (pN), the number of examined LNs (ELN), and the LN ratio (LNR) are widely used to evaluate LN status in patients with GC after gastrectomy. pN is the number of positive regional LNs and represents the N stage in the American Joint Committee on Cancer (AJCC) TNM staging system, which is the most widely used staging system for GC. N stage is categorized according to the number of metastatic regional LNs. In the seventh AJCC staging system, no metastatic LN, 1–2, 3–6, 7–15, and ≥ 16 metastatic regional LNs are classified as N0, N1, N2, N3a, and N3b, respectively. ELN is defined as the number of LNs examined, indicating locoregional disease clearance during gastrectomy, which may influence the accuracy of N staging and even lead to stage migration. ELN is negatively associated with the risk of distant metastasis and positively correlated with prognosis in patients with GC (5–7). The LNR refers to the ratio of the number of metastatic LN to the number of ELN. The LNR is a composite of both pN and ELN and is less affected by clinical stage migration. Thus, the LNR is considered a useful prognostic indicator in patients with GC.

Recent MD Anderson Cancer Center research demonstrated that patients with ELN ≥ 30 and LNR < 0.3 have longer relapse-free survival than those with ELN < 30 and LNR > 0.3, respectively. Using the National Clinical Database, Erstad et al. (8) investigated 22,018 patients with GC with LN metastasis and observed that patients with ELN ≥ 30 had a significant survival advantage. However, neither of the abovementioned studies considered potential competing events against disease-specific death in patients with GC. A competing event refers to an event that precludes or reduces the probability that an event of interest will occur. For instance, cardiac death, suicide, and accidental death will prevent the probability of cancer-specific death. When the study outcome is cancer-specific death, events other than the study outcome are termed competing events. Indeed, when study populations are susceptible to competing events, Cox proportional hazards regression model likely leads to a significant overestimation of the rates of overcoming events. In such cases, a competing risk model (CRM) is recommended (9) because it allows for a more accurate assessment of the association between predictor variables and outcomes than Cox regression model (10–12). A CRM is an analysis method for estimating the cumulative incidence probability of outcome events by using the cumulative incidence function and processing the survival data of outcomes and multiple competing risk events. Gao et al. conducted a study on long-term mortality using Cox proportional hazards analysis and competing risks regression analysis, emphasizing that CRM is more suitable for survival analysis in patients with classical Hodgkin lymphoma (10). Fu et al. found that the errors introduced by the Cox proportional hazards model could be corrected by CRM, thereby providing more accurate survival predictions, when exploring the impact of non-breast cancer-specific mortality on the overall survival of patients with resectable breast cancer (12). Few reports have used a CRM to conduct comparisons among ELN, LNR, and pN.

The prognosis of patients with stage II GC typically lies between early and advanced stages of GC. For patients with LN-positive stage II GC, LN status (such as ELN, LNR, and pN) plays a crucial role in predicting the prognosis (13). However, there is limited in-depth research in the literature on this specific group. Moreover, studies have indicated that patients with GC may experience higher toxicity during postoperative chemotherapy and radiotherapy, especially hematological and gastrointestinal toxicities (14). The patients with LN-positive stage II present with more complex conditions and may face a higher risk of treatment-related non-GCSD events. This makes the influence of competing risk events particularly significant when assessing overall survival, thus requiring a specialized analysis. In addition, in-depth research on LN-related markers (such as ELN, LNR, and pN) and their impact on the prognosis of patients with stage II LN-positive GC can contribute to optimizing the extent of LN dissection and postoperative follow-up strategies while also providing theoretical evidence for personalized treatment. Therefore, using a CRM, the present study aimed to evaluate the prognostic effects of ELN, LNR, and pN in patients with LN-positive stage II GC undergoing resection and chemotherapy. Because a cohort of patients from a single medical center usually has the disadvantages of small sample size, low statistical efficiency, and evident selection bias, we analyzed a large cohort of patients from the Surveillance, Epidemiology, and End Results (SEER) database.

## 2 Materials and methods

### 2.1 Data source

Data were obtained from the SEER database, which is funded by the National Cancer Institute. The publicly available SEER database collects and publishes comprehensive information on cancer incidence, characteristics, and survival in the United States (US). Additionally, the SEER database covers approximately 48% of the US population, including various races (15). The requirement for ethical approval was waived.

**Figure 1:**
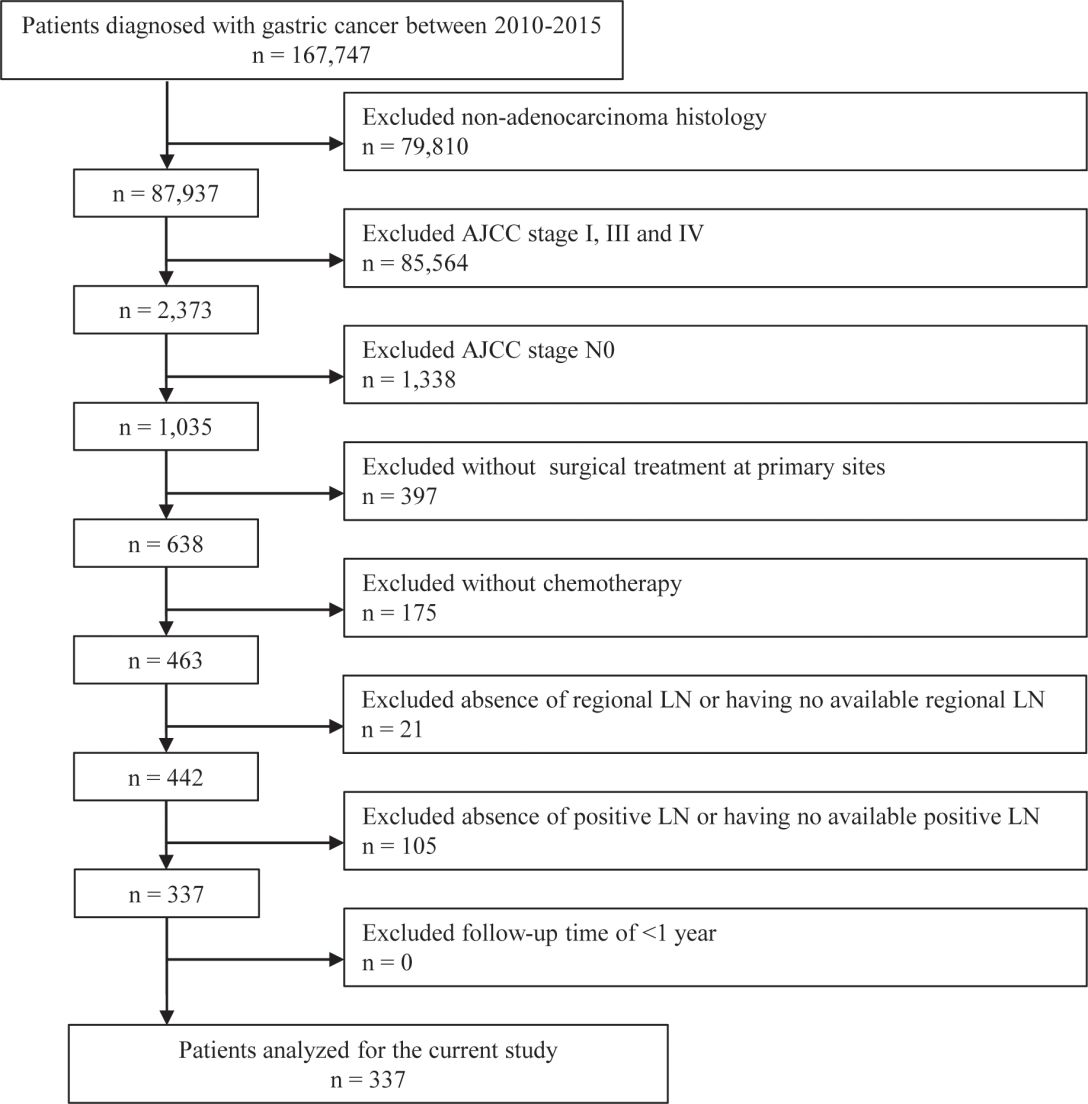
Flow diagram of patient selection. LN, lymph node.

Patients who met the following criteria were enrolled: (i) being diagnosed with GC; (ii) having adenocarcinoma histology; (iii) having AJCC stage II; (iv) having undergone surgical treatment at primary sites; and (v) having received chemotherapy. The exclusion criteria were as follows: (i) AJCC stage N0; (ii) absence of regional LN or having no available regional LN; (iii) absence of pLN or having no available pLN; and (iv) follow-up time of < 1 year. The selection procedure is illustrated in **Figure 1**. Finally, 337 patients with GC were included in the analysis.

### 2.2 Variables

We collected the following data for each patient: age at diagnosis (24–39, 40–59, or 60–88 years), sex (female or male), race (black, white, or other), marital status at the time of diagnosis (divorced/separated, married, single/unmarried, or widowed/other), year of diagnosis (2010, 2011, 2012, 2013, 2014, or 2015), AJCC stage (IIa or IIb according to the seventh AJCC staging system), T stage (T1, T2, or T3), N stage (N1 or N2–N3), radiotherapy (no/not available [NA] or yes), surgical procedure (proximal/distal gastrectomy [PG/DG], total gastrectomy [TG], or others), grade (I–II, III–IV, or unknown), multiple lesions (categorized as no or yes), prior malignancy history (categorized as no or yes), ELN (categorized as ≤ 16 or > 16), LNR (categorized as ≤ 0.1 or > 0.1), pN (categorized as pN1, pN2, or pN3, defined as the number of positive LNs [1–2, 3–6, or ≥ 7]), survival time, and survival outcome (alive, death from GC, death from non-GC causes, or censored). Alive was defined as survival until the end of follow-up. Death due to primary GC was defined as death from GC. Death due to non-GC causes and other secondary primary cancers was defined as death from non-GC causes. Other cases were censored.

### 2.3 Statistical analysis

Demographic and clinicopathological data were collected at baseline, classified as categorical data, described as frequencies and proportions, and then compared using χ^2^ test. ELN, LNR, and pN were described as median and interquartile ranges. Optimal cutoff values for ELN and LNR were determined using restricted cubic splines, and pN was divided into three groups based on the AJCC staging system for further analysis. OS and disease-specific survival (DSS) were assessed and plotted using Kaplan–Meier survival curves. Cox univariate and multivariate regression analyses were performed to identify the effects of ELN, LNR, and pN on OS and DSS, and hazard ratios (HRs) were reported with 95% confidence intervals (CIs). Competing risks of mortality were accommodated and plotted using cumulative incidence curves, while size effects were calculated using sub-distribution HRs (SHRs) with corresponding 95% CIs. A secondary analysis was conducted using the Fine and Gray regression method for competing risk analysis. In Cox regression model and CRM, we conducted a subgroup analysis to test the effects of ELN, LNR, and pN status on outcomes in each subgroup of covariates. Subsequently, interaction tests were conducted to evaluate the interactions between ELN, LNR, or pN and different subgroups. Statistical significance was defined as a two-sided *P*-value < 0.05. All statistical analyses were performed using R software version 4.1.1 (R Foundation for Statistical Computing, Vienna, Austria).

## 3 Results

### 3.1 Clinical characteristics

From 2010 to 2015, the data of 167,747 patients with GC were obtained from the SEER database. Among these patients, 167,410 were excluded due to the histological type, AJCC stage, surgery status, chemotherapy status, number of LNs, or survival times. Finally, 337 patients were included in subsequent analyses during a median follow-up period of 30 months. Among these patients, GC-specific death (GCSD group) occurred in 90 patients, non-GC-specific death (non-GCSD group) was observed in 44 patients, and 203 patients were alive at the end of follow-up (alive group). As shown in **Table 1** for the CRM, the non-GCSD group included more patients diagnosed at 60–88 years of age (89%) than the GCSD (69%) and alive (65%) groups (*P* = 0.02). Statistically significant associations were observed for the year of diagnosis (*P* = 0.03). Moreover, patients in the non-GCSD group tended to have multiple lesions (59%) and prior malignancy history (57%) than those in the GCSD (6% and 0%) and alive (20% and 14%) groups (both *P* < 0.001). With respect to pathological LNs, the median ELN was higher in the alive group (18) than in the GCSD (14.5) and non-GCSD (12) groups (*P* < 0.001), whereas the median LNR was lower in the alive group (0.08) than in the GCSD (0.13) and non-GCSD (0.15) groups (*P* < 0.001). A comparison between the three groups revealed no significant differences in sex, race, marital status at diagnosis, AJCC stage, T stage, N stage, radiotherapy, surgical procedure, grade, and pN. **Supplementary Table 1** presents the clinical characteristics of the normal model. In general, by comparing the death group to the alive group, significant differences were found in the year of diagnosis, ELN, and LNR (*P* < 0.05).

**Table 1:** Baseline clinicopathological characteristics of the enrolled patients for CRM.

Variables	Total (n = 337)	GCSD (n = 90)	non-GCSD (n = 44)	Live (n = 203)	*P* value
Age, n (%)					0.022
24~39	12 (4)	2 (2)	1 (2)	9 (4)	
40~59	92 (27)	26 (29)	4 (9)	62 (31)	
60~88	233 (69)	62 (69)	39 (89)	132 (65)	
Sex, n (%)					0.418
Female	96 (28)	23 (26)	10 (23)	63 (31)	
Male	241 (72)	67 (74)	34 (77)	140 (69)	
Race, n (%)					0.595
White	210 (62)	57 (63)	30 (68)	123 (61)	
Black	70 (21)	16 (18)	10 (23)	44 (22)	
Others	57 (17)	17 (19)	4 (9)	36 (18)	
Marital, n (%)					0.413
Married	204 (61)	52 (58)	29 (66)	123 (61)	
Divorced/Separated	33 (10)	10 (11)	4 (9)	19 (9)	
Single/Unmarried	50 (15)	14 (16)	2 (5)	34 (17)	
Widowed/Others	50 (15)	14 (16)	9 (20)	27 (13)	
Year of diagnosis, n (%)					0.033
2010	42 (12)	18 (20)	5 (11)	19 (9)	
2011	61 (18)	23 (26)	9 (20)	29 (14)	
2012	71 (21)	15 (17)	10 (23)	46 (23)	
2013	53 (16)	12 (13)	10 (23)	31 (15)	
2014	57 (17)	14 (16)	5 (11)	38 (19)	
2015	53 (16)	8 (9)	5 (11)	40 (20)	
AJCC stage, n (%)					0.339
IIa	66 (20)	14 (16)	7 (16)	45 (22)	
IIb	271 (80)	76 (84)	37 (84)	158 (78)	
T stage, n (%)					0.296
T1	79 (23)	15 (17)	12 (27)	52 (26)	
T2	129 (38)	36 (40)	13 (30)	80 (39)	
T3	129 (38)	39 (43)	19 (43)	71 (35)	
N stage, n (%)					0.225
N1	275 (82)	76 (84)	39 (89)	160 (79)	
N2~N3	62 (18)	14 (16)	5 (11)	43 (21)	
Radiotherapy, n (%)					0.465
No/NA	118 (35)	34 (38)	18 (41)	66 (33)	
Yes	219 (65)	56 (62)	26 (59)	137 (67)	
Surgical procedure, n (%)					0.217
TG	70 (21)	25 (28)	11 (25)	34 (17)	
Others	27 (8)	8 (9)	3 (7)	16 (8)	
PG/DG	240 (71)	57 (63)	30 (68)	153 (75)	
Grade, n (%)					0.786
I~II	125 (37)	34 (38)	19 (43)	72 (35)	
III~IV	201 (60)	53 (59)	23 (52)	125 (62)	
Unknown	11 (3)	3 (3)	2 (5)	6 (3)	
Multiple lesions, n (%)					< 0.001
No	266 (79)	85 (94)	18 (41)	163 (80)	
Yes	71 (21)	5 (6)	26 (59)	40 (20)	
Prior malignancy history, n (%)					< 0.001
No	284 (84)	90 (100)	19 (43)	175 (86)	
Yes	53 (16)	0 (0)	25 (57)	28 (14)	
LNR, Median (Q1, Q3)	0.10 (0.06, 0.20)	0.13 (0.07, 0.25)	0.15 (0.08, 0.25)	0.08 (0.06, 0.17)	< 0.001
ELN, Median (Q1, Q3)	16 (10, 23)	14.5 (8, 21.75)	12 (7, 18.25)	18 (12, 24)	< 0.001
pN, Median (Q1, Q3)	2 (1, 2)	2 (1, 2)	2 (1, 2)	1 (1, 2)	0.966

CRM, competing risk model; GCSD, gastric cancer-specific death; n, number; AJCC, American Joint Committee on Cancer; NA, not available; PG/DG, proximal/distal gastrectomy; TG, total gastrectomy; ELN, examined lymph node; pN, positive lymph node; LNR, lymph node ratio; Q1, the 25th percentile of the sample data; Q3, the 75th percentile of the sample data.

### 3.2 Association between patient characteristics and outcomes using the normal model

**Figure 2:**
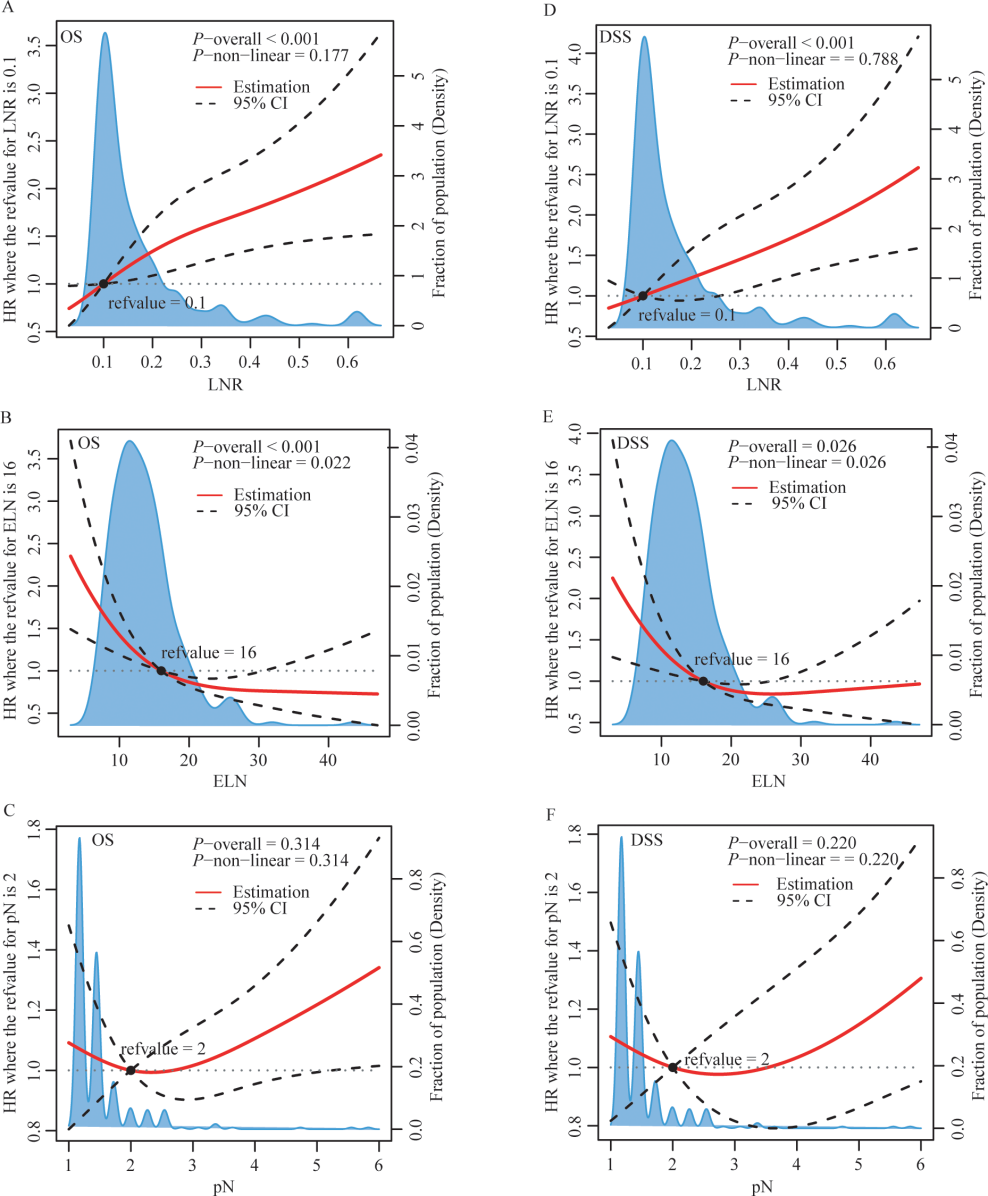
| Restricted cubic splines of LNR **(A, D)**, ELN **(B, E)** and pN **(C, F)** for OS **(A-C)** and DSS **(D-F).** HR, hazard ratio; CI, confidence interval; LNR, lymph node ratio; ELN, examined lymph node; pN, positive lymph node.

**Figure 3:**
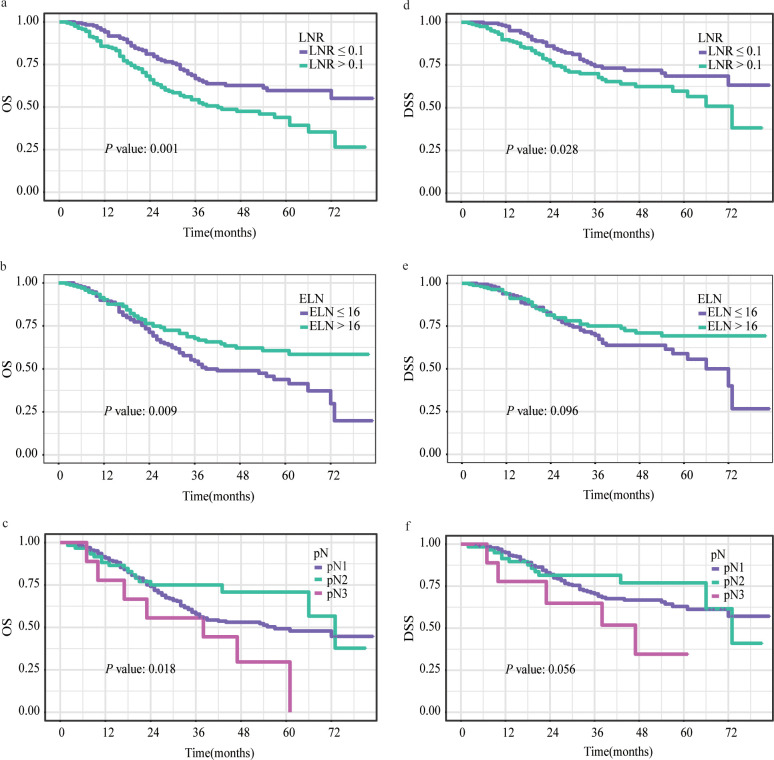
Kaplan–Meier survival curves for OS **(A-C)** and DSS **(D-F)** stratified by LNR **(A, D)**, ELN **(B, E)** and pN **(C, F)**. OS, overall survival; DSS, disease-specific survival; LNR, lymph node ratio; ELN, examined lymph node; pN, positive lymph node.

Based on the restricted cubic splines, optimal cutoff values for ELN and LNR were 16 and 0.1 for further study (**Figure 2**). While pN was categorized as pN1, pN2, or pN3, defined as the number of positive LNs (1–2, 3–6, or ≥ 7) based on the AJCC staging system for further study. **Figure 3**, **Supplementary Figures 1**, **2** show Kaplan–Meier curves for OS and DSS in the normal model analysis and models stratified according to covariates, including age, sex, race, marital status at diagnosis, year of diagnosis, AJCC stage, T stage, N stage, radiotherapy, surgical procedure, grade, multiple lesions, prior malignancy history, ELN, pN, and LNR. As shown in **Figures 3A, D**, the patients with LN-positive stage II GC with LNR ≤ 0.1 had significantly superior OS and DSS compared to those with LNR > 0.1. As shown in **Figure 3B**, patients with an ELN > 16 had a significantly greater OS benefit than those with an ELN ≤ 16. The impact of pN on the OS of patients with LN-positive stage II GC varied extensively with different pN stages (**Figure 3C**). However, ELN > 16 and pN had no significant effect on DSS (**Figures 2E, F**). Regarding OS and DSS, similar findings were observed for patients with different surgical procedures and different years of diagnosis (**Supplementary Figures 1**, **2**). Additionally, the results showed that age, race, sex, and marital status at the time of diagnosis had no significant impact on OS and DSS in patients with LN-positive stage II GC (**Supplementary Figures 1**, **2**).

We subsequently performed Cox univariate and multivariate regression analyses to explore the prognostic factors for patients with LN-positive stage II GC. In univariate Cox regression analysis, compared to ELN ≥ 16, ELN <16 showed a significant correlation with OS (HR = 0.63, 95% CI: 0.45-0.90, *P* = 0.01); however, there was no significant association with DSS (HR = 0.70, 95% CI: 0.46-1.07, *P* = 0.10). Patients with LNR > 0.1 had worse OS (HR = 1.79, 95% CI: 1.27-2.53, *P* < 0.001) and DSS (HR = 1.59, 95% CI: 1.05-2.42, *P* = 0.03) than those with LNR ≤ 0.1. In contrast, compared to pN1, neither pN2 nor pN3 had a statistical relationship with OS or DSS (both *P* > 0.05) (**Tables 2**, **3**).

**Table 2:** Cox univariate and multivariate regression analyses of clinicopathological characteristics associated with OS.

Characteristics	Univariate analysis			Multivariate analysis								
				LNR			ELN			pN		
	HR	95% CI	*P* value	HR	95% CI	*P* value	HR	95% CI	*P* value	HR	95% CI	*P* value
Age												
24~39	Reference			Reference			Reference			Reference		
40~59	1.342	0.409-4.397	0.627	1.212	0.341-4.302	0.766	1.192	0.340-4.182	0.784	1.076	0.306-3.778	0.909
60~88	1.869	0.593-5.893	0.286	1.592	0.471-5.378	0.454	1.636	0.488-5.486	0.425	1.524	0.455-5.100	0.494
Sex												
Female	Reference			Reference			Reference			Reference		
Male	1.246	0.841-1.847	0.273	1.338	0.840-2.132	0.220	1.324	0.838-2.090	0.229	1.470	0.923-2.341	0.104
Race												
White	Reference			Reference			Reference			Reference		
Black	0.891	0.574-1.382	0.605	0.857	0.509-1.445	0.563	0.880	0.524-1.480	0.631	0.853	0.510-1.427	0.545
Others	0.824	0.511-1.327	0.425	0.711	0.423-1.194	0.197	0.775	0.465-1.291	0.328	0.769	0.459-1.290	0.320
Marital status												
Married	Reference			Reference			Reference			Reference		
Divorced/Separated	1.037	0.588-1.830	0.900	1.067	0.575-1.980	0.837	1.243	0.673-2.294	0.487	1.225	0.661-2.269	0.519
Single/Unmarried	0.779	0.456-1.332	0.362	0.918	0.505-1.668	0.779	0.935	0.520-1.681	0.823	0.889	0.493-1.602	0.695
Widowed/Others	1.162	0.731-1.846	0.526	1.387	0.823-2.335	0.219	1.298	0.776-2.169	0.321	1.267	0.750-2.140	0.377
Year of diagnosis												
2010	Reference			Reference			Reference			Reference		
2011	1.228	0.707-2.130	0.466	1.071	0.592-1.937	0.820	1.098	0.613-1.968	0.753	1.220	0.672-2.217	0.513
2012	0.807	0.449-1.451	0.474	0.820	0.440-1.526	0.530	0.819	0.441-1.524	0.529	0.916	0.487-1.725	0.787
2013	1.202	0.655-2.206	0.553	1.072	0.558-2.058	0.835	1.025	0.538-1.955	0.939	1.154	0.598-2.229	0.669
2014	1.202	0.634-2.279	0.573	1.337	0.671-2.665	0.409	1.167	0.589-2.314	0.658	1.325	0.656-2.680	0.433
2015	1.664	0.802-3.450	0.171	1.760	0.821-3.776	0.146	1.688	0.789-3.611	0.177	1.887	0.871-4.090	0.107
AJCC stage												
IIa	Reference			Reference			Reference			Reference		
IIb	1.388	0.871-2.212	0.168	1.102	0.641-1.892	0.726	1.136	0.660-1.954	0.646	0.994	0.575-1.718	0.982
T stage												
T1	Reference			Reference			Reference			Reference		
T2	1.090	0.681-1.744	0.720	0.925	0.562-1.521	0.758	1.039	0.637-1.693	0.879	1.115	0.669-1.856	0.677
T3	1.395	0.883-2.203	0.154	1.180	0.663-2.099	0.573	1.334	0.761-2.336	0.314	1.457	0.826-2.570	0.194
N stage												
N1	Reference			Reference			Reference			Reference		
N2~N3	0.739	0.455-1.200	0.221	0.569	0.313-1.032	0.063	0.945	0.539-1.656	0.844	0.968	0.260-3.601	0.962
Radiotherapy												
No/NA	Reference			Reference			Reference			Reference		
Yes	0.727	0.513-1.029	0.072	0.708	0.489-1.024	0.067	0.733	0.507-1.058	0.097	0.752	0.519-1.088	0.131
Surgical procedure												
TG	Reference			Reference			Reference			Reference		
Others	0.706	0.359-1.388	0.313	0.644	0.303-1.367	0.252	0.679	0.327-1.409	0.299	0.637	0.306-1.329	0.230
PG/DG	0.629	0.426-0.928	0.019	0.696	0.460-1.052	0.085	0.710	0.472-1.069	0.101	0.700	0.464-1.055	0.088
Grade												
I~II	Reference			Reference			Reference			Reference		
III~IV	0.958	0.674-1.361	0.809	0.882	0.604-1.288	0.517	0.862	0.591-1.256	0.438	0.853	0.583-1.248	0.412
Unknown	1.147	0.458-2.870	0.770	1.011	0.388-2.636	0.982	1.099	0.425-2.840	0.846	1.121	0.433-2.902	0.814
Multiple lesions												
No	Reference			Reference			Reference			Reference		
Yes	1.099	0.735-1.642	0.645	0.579	0.242-1.387	0.220	0.635	0.268-1.500	0.300	0.515	0.211-1.257	0.145
Prior malignancy history												
No	Reference			Reference			Reference			Reference		
Yes	1.363	0.882-2.106	0.163	1.710	0.655-4.459	0.273	1.625	0.628-4.204	0.316	2.147	0.803-5.737	0.128
LNR												
LNR ≤ 0.1	Reference			Reference			Not applicable			Not applicable		
LNR > 0.1	1.788	1.265-2.527	< 0.001	2.225	1.515-3.269	< 0.001						
ELN												
ELN ≤ 16	Reference			Not applicable			Reference			Not applicable		
ELN > 16	0.633	0.447-0.896	0.010				0.670	0.465-0.966	0.032			
pN												
pN1	Reference			Not applicable			Not applicable			Reference		
pN2	0.686	0.412-1.144	0.148							0.822	0.224-3.012	0.767
pN3	1.925	0.896-4.137	0.093							2.803	1.018-7.715	0.046

OS, overall survival; HR, hazard ratio; CI, confidence interval; LNR, lymph node ratio; ELN, examined lymph node; pN, positive lymph node; AJCC, American Joint Committee on Cancer; NA, not available; TG, total gastrectomy; PG/DG, proximal/distal gastrectomy.

**Table 3:** Cox univariate and multivariate regression analyses of clinicopathological characteristics associated with DSS.

Characteristics	Univariate analysis			Multivariate analysis								
				LNR			ELN			pN		
	HR	95% CI	*P* value	HR	95% CI	*P* value	HR	95% CI	*P* value	HR	95% CI	*P* value
Age												
24~39	Reference			Reference			Reference			Reference		
40~59	1.755	0.416-7.396	0.443	1.485	0.318-6.926	0.615	1.482	0.322-6.829	0.614	1.405	0.304-6.493	0.663
60~88	1.732	0.423-7.082	0.445	1.796	0.403-7.997	0.442	1.830	0.414-8.088	0.425	1.800	0.408-7.935	0.437
Sex												
Female	Reference			Reference			Reference			Reference		
Male	1.181	0.735-1.897	0.492	1.248	0.711-2.193	0.440	1.253	0.721-2.178	0.424	1.350	0.771-2.364	0.293
Race												
White	Reference			Reference			Reference			Reference		
Black	0.841	0.482-1.466	0.541	0.701	0.354-1.387	0.307	0.708	0.359-1.398	0.320	0.722	0.370-1.412	0.341
Others	1.027	0.597-1.768	0.923	0.898	0.491-1.644	0.728	0.974	0.537-1.767	0.931	0.974	0.534-1.777	0.932
Marital status												
Married	Reference			Reference			Reference			Reference		
Divorced/Separated	1.148	0.583-2.261	0.689	1.020	0.491-2.122	0.957	1.180	0.569-2.446	0.656	1.106	0.532-2.300	0.788
Single/Unmarried	1.056	0.585-1.906	0.856	1.116	0.569-2.190	0.749	1.167	0.599-2.274	0.649	1.036	0.534-2.010	0.917
Widowed/Others	1.096	0.607-1.978	0.761	1.142	0.588-2.220	0.695	1.067	0.556-2.048	0.846	1.038	0.537-2.005	0.912
Year of diagnosis												
2010	Reference			Reference			Reference			Reference		
2011	1.163	0.614-2.205	0.642	1.114	0.568-2.184	0.754	1.148	0.585-2.252	0.689	1.238	0.626-2.447	0.539
2012	0.639	0.314-1.302	0.217	0.641	0.303-1.356	0.245	0.656	0.310-1.389	0.271	0.714	0.334-1.525	0.384
2013	0.873	0.408-1.865	0.725	0.747	0.334-1.670	0.478	0.767	0.344-1.708	0.515	0.849	0.378-1.911	0.693
2014	1.148	0.547-2.409	0.715	1.395	0.629-3.095	0.413	1.258	0.566-2.797	0.573	1.359	0.602-3.068	0.461
2015	1.358	0.555-3.320	0.502	1.526	0.613-3.804	0.364	1.519	0.608-3.797	0.371	1.720	0.678-4.360	0.253
AJCC stage												
IIa	Reference			Reference			Reference			Reference		
IIb	1.405	0.794-2.487	0.243	1.003	0.517-1.946	0.993	1.088	0.561-2.111	0.803	0.967	0.496-1.883	0.920
T stage												
T1	Reference			Reference			Reference			Reference		
T2	1.433	0.784-2.619	0.242	1.240	0.657-2.341	0.507	1.357	0.725-2.540	0.340	1.435	0.746-2.760	0.280
T3	1.696	0.934-3.080	0.083	1.696	0.802-3.590	0.167	1.802	0.867-3.748	0.115	1.970	0.936-4.142	0.074
N stage												
N1	Reference			Reference			Reference			Reference		
N2~N3	0.827	0.468-1.463	0.515	0.686	0.336-1.400	0.300	1.108	0.560-2.191	0.768	1.003	0.221-4.544	0.997
Radiotherapy												
No/NA	Reference			Reference			Reference			Reference		
Yes	0.761	0.496-1.166	0.209	0.752	0.473-1.195	0.228	0.788	0.498-1.249	0.311	0.806	0.509-1.277	0.359
Surgical procedure												
TG	Reference			Reference			Reference			Reference		
Others	0.729	0.328-1.620	0.438	0.800	0.331-1.936	0.621	0.827	0.348-1.963	0.667	0.722	0.300-1.737	0.467
PG/DG	0.590	0.369-0.945	0.028	0.636	0.385-1.049	0.077	0.663	0.403-1.091	0.106	0.670	0.406-1.105	0.117
Grade												
I~II	Reference			Reference			Reference			Reference		
III~IV	1.041	0.676-1.604	0.854	0.948	0.591-1.520	0.823	0.950	0.592-1.522	0.830	0.924	0.577-1.480	0.742
Unknown	1.085	0.333-3.536	0.892	0.945	0.269-3.319	0.930	1.071	0.310-3.703	0.914	1.229	0.357-4.232	0.744
Multiple lesions												
No	Reference			Reference			Reference			Reference		
Yes	0.214	0.087-0.527	< 0.001	0.169	0.067-0.426	< 0.001	0.180	0.072-0.451	< 0.001	0.178	0.071-0.448	< 0.001
LNR												
LNR ≤ 0.1	Reference			Reference			Not applicable			Not applicable		
LNR > 0.1	1.591	1.048-2.417	0.029	2.084	1.299-3.343	0.002						
ELN												
ELN ≤ 16	Reference			Not applicable			Reference			Not applicable		
ELN > 16	0.702	0.461-1.069	0.099				0.645	0.411-1.013	0.057			
pN												
pN1	Reference			Not applicable			Not applicable			Reference		
pN2	0.805	0.446-1.454	0.472							0.919	0.208-4.058	0.912
pN3	2.135	0.861-5.295	0.102							3.020	0.986-9.249	0.053

DSS, disease-specific survival; HR, hazard ratio; CI, confidence interval; LNR, lymph node ratio; ELN, examined lymph node; pN, positive lymph node; AJCC, American Joint Committee on Cancer; NA, not available; TG, total gastrectomy; PG/DG, proximal/distal gastrectomy.

As indicated by the results of multivariate Cox regression analysis, LNR > 0.1 was an independent factor for shorter OS (HR = 2.23, 95% CI: 1.52-3.27, *P* < 0.001) and DSS (HR = 2.08, 95% CI: 1.30-3.34, *P* = 0.002), whereas ELN > 16 was independently associated with better OS (HR = 0.67, 95% CI: 0.47-0.97, *P* = 0.03); however, there was no significant difference in DSS (HR = 0.65, 95% CI: 0.41-1.01, *P* = 0.06). Compared to patients with pN1, those with pN3 had worse OS (HR = 2.80, 95% CI: 1.02-7.72, *P* = 0.046) but did not show a relationship with DSS (HR = 3.02, 95% CI: 0.99-9.25, *P* = 0.05). Detailed information on the univariate and multivariate regression analyses for OS and DSS is presented in **Tables 2**, **3**.

We then conducted subgroup analyses and interaction tests to evaluate the relationship of the LN status with the OS and DSS in various subgroups in patients with LN-positive stage II GC. The subgroup analysis revealed that in the male subgroup (sex variable), ELN > 16 had a protective effect on OS (HR = 0.62, 95% CI: 0.41-0.94, *P* = 0.02), whereas LNR > 0.1 (HR = 1.98, 95% CI: 1.32-2.97, *P* < 0.001) and pN3 (HR = 2.89, 95% CI: 1.17-7.11, *P* = 0.02) showed the opposite effect. In contrast, neither ELN > 16, LNR > 0.1, nor pN3 were significantly associated with OS in the female subgroup (**Supplementary Table 2**).

As presented in **Supplementary Tables 2**, **3**, LNR > 0.1 showed decreased OS for stages T1 (HR = 2.34, 95% CI: 1.04-5.24, *P* = 0.04) and T3 (HR = 2.13, 95% CI: 1.26-3.62, *P* = 0.005) but not stage T2. LNR > 0.1 showed a decreased DSS (HR = 3.29, 95% CI: 1.03-10.47, *P* = 0.04) for the stage T1 subtype but not stages T2 and T3. Regarding the different ELN and pN groups, no significant difference in OS or DSS was observed for each T stage subgroup.

The tests for interactions were not significant in the subgroup analysis, except for the ELN in the single/unmarried subgroup for OS (HR = 0.31, 95% CI: 0.10-0.97, *P* = 0.045) and the LNR in the year of diagnosis in the 2013 subgroup for OS (HR = 0.25, 95% CI: 0.07-0.84, *P* = 0.03) (**Supplementary Tables 2**, **3**).

### 3.3 Association between patient characteristics and outcomes using the competing risk model

A CRM was used to further investigate the association between patient characteristics and outcomes of patients with LN-positive stage II GC according to the risk of GCSD relative to overall mortality.

**Figure 4:**
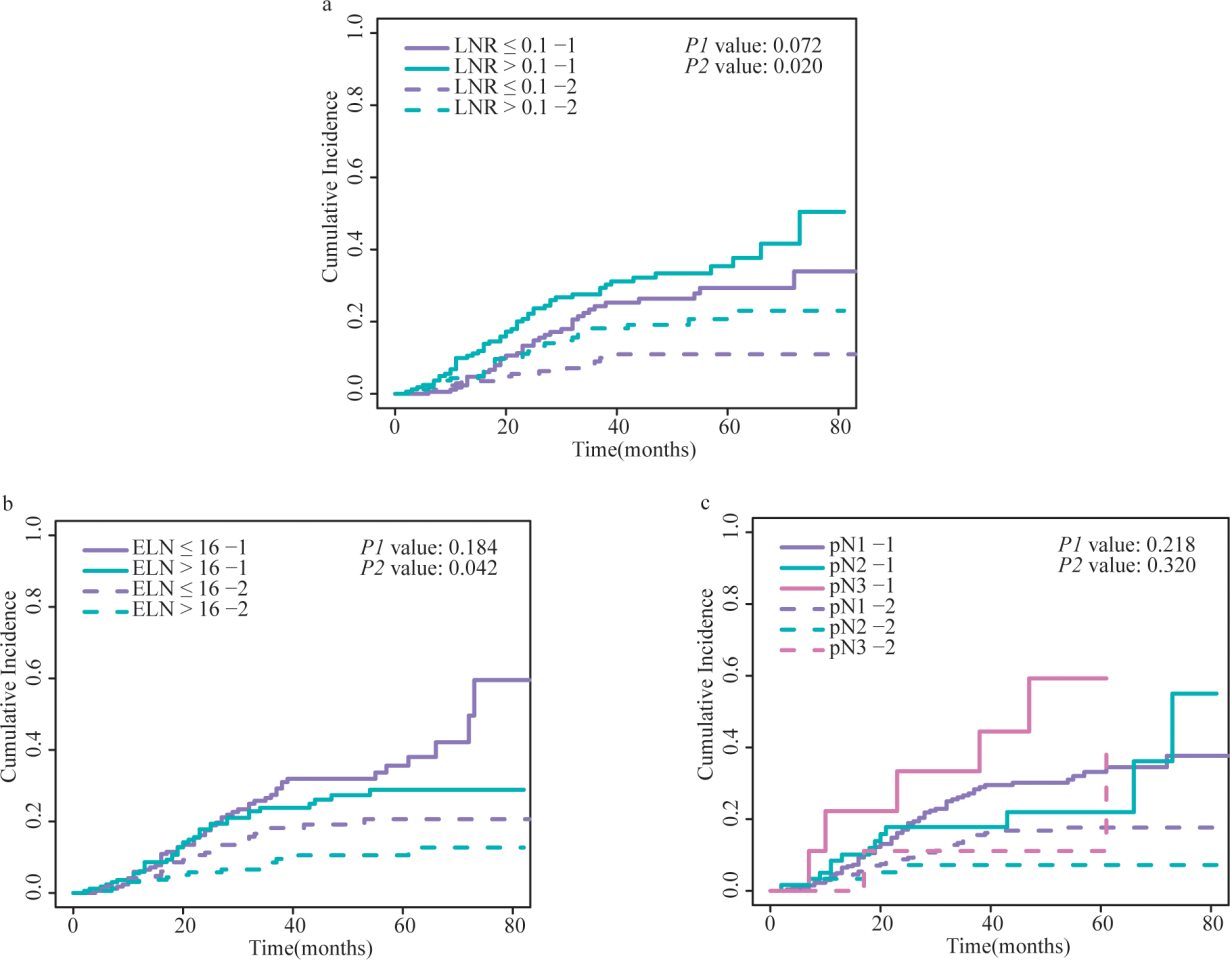
Cumulative incidence of GCSD and non-GCSD stratified by **(A)** LNR, **(B)** ELN and **(C)** pN. The *P1* value represents the statistical result of comparing the cumulative incidence curves of GCSD between different groups using the Fine and Gray competing risks model. The *P2* value represents the comparison of cumulative incidence curves for non-GCSD between different groups. GCSD, gastric cancer-specific death; LNR, lymph node ratio; ELN, examined lymph node; pN, positive lymph node.

The cumulative incidence curves for all variables are shown in **Figure 4** and **Supplementary Figure 3**. Patients with LNR > 0.1 had more non-GCSD than those with LNR ≤ 0.1 (*P* = 0.02), but the cumulative incidences of GCSD were not significantly different (*P* = 0.072). There was a higher cumulative incidence of non-GCSD in patients with ELN ≤ 16 than in those with ELN > 16 (*P* = 0.04); however, the cumulative incidences of GCSD were not significantly different (*P* = 0.18). No statistically significant differences were detected for pN in the cumulative incidence of either GCSD or non-GCSD.

We then performed a Fine and Gray regression analysis to explore the prognostic factors for patients with LN-positive stage II GC in a competing risk regression model.

Based on the results of the multivariate analysis, LNR > 0.1 was identified as an independent factor for shorter DSS (SHR = 1.89, 95% CI: 1.17-3.05, *P* = 0.001), whereas pN3 was independently associated with a lower DSS risk (SHR = 2.80, 95% CI: 1.31-5.97, *P* = 0.01). Compared with patients with ELN ≤ 16, those with ELN > 16 showed no relationship with DSS (SHR = 0.68, 95% CI: 0.42-1.10, *P* = 0.11).

Detailed information regarding the competing risk regression analysis for DSS is presented in **Table 4**.

**Table 4:** Univariate and multivariate competing risk analyses of clinicopathological characteristics associated with DSS.

Characteristics	Univariate analysis			Multivariate analysis								
				LNR			ELN			pN		
	SHR	95% CI	*P* value	SHR	95% CI	*P* value	SHR	95% CI	*P* value	SHR	95% CI	*P* value
Age												
24~39	Reference			Reference			Reference			Reference		
40~59	1.745	0.421-7.240	0.44	1.522	0.362-6.391	0.57	1.513	0.346-6.607	0.58	1.438	0.329-6.281	0.63
60~88	1.558	0.385-6.311	0.53	1.715	0.419-7.013	0.45	1.744	0.407-7.478	0.45	1.733	0.409-7.338	0.46
Sex												
Female	Reference			Reference			Reference			Reference		
Male	1.138	0.707-1.832	0.59	1.295	0.699-2.400	0.41	1.297	0.715-2.353	0.39	1.359	0.737-2.507	0.33
Race												
White	Reference			Reference			Reference			Reference		
Black	0.839	0.488-1.443	0.53	0.646	0.317-1.317	0.23	0.649	0.326-1.294	0.22	0.663	0.339-1.297	0.23
Others	1.101	0.649-1.870	0.72	0.913	0.506-1.648	0.76	0.974	0.551-1.721	0.93	0.989	0.558-1.753	0.97
Marital status												
Married	Reference			Reference			Reference			Reference		
Divorced/Separated	1.163	0.596-2.272	0.66	0.958	0.439-2.088	0.91	1.093	0.511-2.339	0.82	1.051	0.487-2.267	0.90
Single/Unmarried	1.153	0.648-2.052	0.63	1.191	0.592-2.397	0.62	1.238	0.622-2.463	0.54	1.117	0.556-2.244	0.76
Widowed/Others	1.075	0.592-1.954	0.81	1.099	0.524-2.303	0.80	1.043	0.509-2.135	0.91	1.024	0.498-2.106	0.95
Year of diagnosis												
2010	Reference			Reference			Reference			Reference		
2011	1.071	0.583-1.969	0.82	1.115	0.578-2.149	0.75	1.133	0.581-2.209	0.71	1.175	0.604-2.283	0.64
2012	0.585	0.299-1.142	0.12	0.604	0.294-1.238	0.17	0.615	0.297-1.272	0.19	0.635	0.309-1.305	0.22
2013	0.734	0.357-1.511	0.40	0.683	0.311-1.498	0.34	0.703	0.324-1.528	0.37	0.761	0.347-1.667	0.49
2014	0.966	0.494-1.890	0.92	1.31	0.617-2.785	0.48	1.206	0.560-2.595	0.63	1.273	0.587-2.758	0.54
2015	0.998	0.438-2.277	1.00	1.124	0.463-2.729	0.80	1.125	0.460-2.753	0.80	1.259	0.512-3.097	0.62
AJCC stage												
IIa	Reference			Reference			Reference			Reference		
IIb	1.37	0.793-2.366	0.26	0.968	0.509-1.842	0.92	1.051	0.547-2.022	0.88	0.955	0.491-1.857	0.89
T stage												
T1	Reference			Reference			Reference			Reference		
T2	1.496	0.828-2.700	0.18	1.258	0.657-2.409	0.49	1.351	0.726-2.513	0.34	1.415	0.731-2.739	0.30
T3	1.696	0.943-3.050	0.078	1.75	0.833-3.674	0.14	1.846	0.915-3.724	0.09	1.994	0.983-4.047	0.06
N stage												
N1	Reference			Reference			Reference			Reference		
N2~N3	0.853	0.482-1.510	0.58	0.776	0.381-1.580	0.48	1.174	0.591-2.335	0.65	0.947	0.261-3.441	0.93
Radiotherapy												
No/NA	Reference			Reference			Reference			Reference		
Yes	0.792	0.516-1.214	0.28	0.731	0.453-1.179	0.20	0.773	0.485-1.233	0.28	0.804	0.503-1.284	0.36
Surgical procedure												
TG	Reference			Reference			Reference			Reference		
Others	0.778	0.356-1.702	0.53	0.883	0.363-2.152	0.78	0.896	0.383-2.098	0.80	0.794	0.328-1.925	0.61
PG/DG	0.625	0.393-0.993	0.047	0.678	0.401-1.149	0.15	0.699	0.416-1.175	0.18	0.705	0.417-1.193	0.19
Grade												
I~II	Reference			Reference			Reference			Reference		
III~IV	1.045	0.686-1.593	0.84	0.939	0.580-1.520	0.80	0.951	0.590-1.533	0.84	0.915	0.568-1.474	0.71
Unknown	1.019	0.309-3.360	0.98	0.767	0.203-2.899	0.70	0.88	0.249-3.116	0.84	0.99	0.316-3.100	0.99
Multiple lesions												
No	Reference			Reference			Reference			Reference		
Yes	0.173	0.071-0.422	< 0.001	0.135	0.051-0.359	< 0.001	0.144	0.056-0.371	< 0.001	0.145	0.057-0.374	< 0.001
LNR												
LNR ≤ 0.1	Reference			Reference			Not applicable			Not applicable		
LNR > 0.1	1.457	0.964-2.201	0.07	1.888	1.167-3.054	0.01						
ELN												
ELN ≤ 16	Reference			Not applicable			Reference			Not applicable		
ELN > 16	0.758	0.499-1.153	0.20				0.679	0.421-1.096	0.11			
pN												
pN1	Reference			Not applicable			Not applicable			Reference		
pN2	0.841	0.465-1.520	0.57							1.047	0.284-3.855	0.94
pN3	2.085	0.861-5.049	0.10							2.798	1.311-5.970	0.01

DSS: disease-specific survival; SHR: sub-distribution hazard ratio; CI: confidence interval; LNR: lymph node ratio; ELN: examined lymph node; pN: positive lymph node; AJCC: American Joint Committee on Cancer; NA: not available; TG: total gastrectomy; PG/DG: proximal/distal gastrectomy.

We subsequently conducted subgroup analyses and interaction tests to determine the correlation between LN status and DSS in patients with LN-positive stage II GC within various subgroups in the competing risk regression model. The subgroup analysis revealed that in the male subgroup (sex variable), LNR > 0.1 had an adverse effect on DSS (SHR = 1.71, 95% CI: 1.05-2.77, *P* = 0.03), and pN3 showed a similar effect (SHR = 3.18, 95% CI: 1.13-8.97, *P* = 0.03). Neither ELN > 16, LNR > 0.1, nor pN3 were significantly associated with DSS in the female subgroup (**Supplementary Table 4**).

## 4 Discussion

In this large retrospective study, we focused on the LN status (particularly ELN, LNR, and pN) in patients with LN-positive stage II GC undergoing resection and chemotherapy. This study had three main findings. First, the ELN, LNR, and pN were all independently associated with OS, and only LNR was independently associated with DSS. Second, when adjusted for the CRM, the LNR and pN maintained prognostic value beyond ELN. Third, the prognostic value of pN for DSS was likely to be influenced by death from other causes (competing events). LNR, in contrast, demonstrated stronger robustness in predicting DSS for patients with LN-positive stage II GC than ELN and pN. Thus, LNR may serve as a reliable and useful prognostic factor in patients with GC.

Using multivariate Cox analysis, we confirmed that pN3 was independently associated with poor OS, but not DSS. However, we found that pN3 was a prognostic factor for DSS when the competing risks were considered. Currently, the TNM classification is the most widely used staging system worldwide to predict prognosis and guide treatment strategies. pN stage is one of the most important prognostic indicators in patients with GC. A previous study showed that pN was an independent predictor of OS in patients with GC after curative-intent gastrectomy (16), and another study reported that pN stage was associated with OS after curative surgery in patients with LN-positive GC (17). Conversely, other cases have discussed that pN may be inappropriate and insufficient to stage and predict prognosis (18). Indeed, a previous study showed that the N3b stage had a better OS than N2 and N3a stages in patients with M0 GC (19). Moreover, Qian et al. (20) demonstrated that the pN stage was not independently associated with OS, which is consistent with the findings of Kulig et al. (21) Additionally, stage migration is another condition; this means that the number of pNs depends on the number of LN dissections. Therefore, an adequate marker for staging and meticulous prediction of prognosis is required.

Numerous studies have attempted to assess the prognostic significance and optimal number of ELN in patients with GC. A recent study demonstrated that ELN was independently associated with OS in patients with GC (8). Studies have also demonstrated that patients with GC with an ELN ≥ 16 have better survival outcomes than those with an ELN < 16 (22, 23). In the present study, Multivariate Cox analysis confirmed that ELN > 16 persisted in being independently related to superior OS. Additionally, both the National Comprehensive Cancer Network (24) and AJCC (25) committees recommend the assessment of at least 16 LNs for precise staging and accurate prognostic evaluation. Nevertheless, Wong et al. (26) showed that ELN was not significantly correlated with survival. Thus, it is worth further evaluating the optimal number of ELN and the relationship between ELN and prognosis in GC to guide clinical practice.

The results of the CRM demonstrated that patients with an ELN > 16 experienced less non-GCSD but did not have lower cumulative GCSD rates. Moreover, an ELN > 16 was still not a prognostic factor for DSS when competing risks were considered; this indicated that patients with ELN > 16 benefited from better OS likely because of less non-GCSD. We speculated that patients with ELN ≤ 16 may present with comorbidities that compel surgeons to retrieve a limited number of LNs. Non-GCSD from comorbidities may lead to poor prognosis in patients with ELN ≤ 16. A previous study also suggested that comorbidity is a risk factor for survival after gastrectomy (27). This risk should be carefully considered in the subsequent treatment of patients with GC and comorbidities.

Multivariate Cox analysis confirmed that LNR > 0.1 was an independent unfavorable factor for DSS and OS. Many recent studies have emphasized the importance of LNR as a prognostic factor in patients with GC. Both a large cohort study (28) and an integrated analysis (29) revealed that LNR could reliably and accurately predict survival in patients with GC. A recent study also demonstrated that a high LNR was associated with worse OS in patients with GC (30). Similar results were found in patients with GC after curative resection (31), patients with GC with LN metastasis (32), and patients with pathological Stage II/III GC (13). Additionally, LNR was a significant and independent predictor of prognosis regardless of the pathological stage (33) and the number of resected nodes (34) in patients with GC. Nevertheless, previous studies did not conduct competing risk analyses to evaluate the relationship between the LNR and prognosis. Based on our results, LNR > 0.1 was still an independent unfavorable factor for DSS when competing risks were considered, indicating that an LNR > 0.1 is a valuable prognostic indicator for GC that is of great value in clinical practice.

Aoyama et al. demonstrated that the prognostic value of LNR was higher in patients with pN3 stage disease than in those with pN1 or pN2 stage disease (30). Similar results were observed in another study by Hung et al. (35), who found that a higher LNR was independently related to inferior OS in patients with stage N3b GC. The abovementioned results were inconsistent with our findings, showing that LNR > 0.1 was an unfavorable factor for both DSS and OS in the cN1 subgroup but had no significant impact in the cN2 or cN3 subgroups. Interestingly, Wu et al. (36) demonstrated contradictory results, showing that LNR was an independent prognostic factor only in patients with stage III GC but not in those with stage I, II, and IV. In the present study, we demonstrated the promising prognostic value of LNR in patients with stage II GC.

These differences may be explained as follows. First, in view of the patients’ background, all patients in the studies by Aoyama et al. (30), Hung et al. (35), and Wu et al. (36) were from Asia (Japan, Taiwan, and China, respectively); in contrast, we evaluated patients from the US. Additionally, we enrolled more older patients than the other three studies. Furthermore, we did not evaluate patients with T4 stage GC, whereas the other studies did. Second, the number of retrieved LNs and the number of metastatic LNs were different. The median number of retrieved LNs was 31, 43, 22, and 16, while the median number of metastatic LNs was 7.7, 22, 4, and 2 in the studies by Aoyama et al. (30), Hung et al. (35), Wu et al. (36), and our study, respectively. The LNR may be affected by the number of retrieved and metastatic LNs. Third, the cut-off values of the LNR were different. We set the cut-off value at 0.1 using the restricted cubic splines; Aoyama et al. (30) set the cut-off value of the LNR to 0.23, based on the 3- and 5-year OS rates; Hung et al. (35) set a cut-off value of 0.8; and Wu et al. (36) stratified the cut-off value of the LNR into 0, 0–0.2, 0.2–0.5, and > 0.5, based on previous studies (37). Hence, the LNR may be more suitable under certain conditions, and a standardized LNR classification for GC should be further investigated.

Over the last few years, the LNR, an alternative LN staging system, has emerged as superior to pN and ELN in predicting the prognosis of GC. Previous studies have shown that the LNR predicts OS more powerful than N stage (38, 39) and pN (40). Additionally, the LNR maintained its effectiveness superior to the pN stage regardless of ELN (41, 42). LNR was an independent prognostic factor in patients with GC, whereas pN and ELN were not (43, 44). Additionally, the LNR remains an independent prognostic factor for OS when ELN < 16 rather than pN (45). Our results also demonstrated that the LNR has a better prognostic value than the ELN and pN. Furthermore, Huang et al. combined the LNR with the AJCC staging system to construct a new LNR-based AJCC (rAJCC) staging system for GC. The authors found that the rAJCC stratified patients better than the AJCC staging system (46). In contrast, the pN stage cannot distinguish the prognostic discrepancy between different LNR groups (47). Therefore, the above results suggest that the LNR provides greater prognostic validity than the pN or ELN. The mechanism contributing to the different effects of the LNR and pN on GC prognosis may be explained as follows. First, the LNR is only a ratio and is not affected by the ELN, which compensates for the stage migration (19). However, as there is a significant correlation between the pN and ELN (45), the LNR may be more useful even if the ELN is small. Second, the LNR is not affected by how the surgery or field of LN dissection is performed (29). Third, LNR exhibits a relationship with the immune response defense mechanism against cancer metastasis (33, 48).

The univariate and multivariate analyses showed no significant association between several factors (such as age, sex, AJCC stage, and T stage) and DSS. However, these factors are often considered important prognostic indicators in GC. Several reasons can be speculated for these results. In this specific population (patients with LN-positive stage II GC undergoing resection and chemotherapy), all patients were AJCC stage II (including IIa and IIb), and both T and N stages had relatively narrow ranges. As a result, these factors (such as age, sex, AJCC stage, and T stage) were unable to further distinguish the prognosis of patients in the present study. Additionally, the limited sample size may have reduced the statistical significance of these variables and the DSS. It is important to note that this does not imply that these factors lack prognostic significance in the overall GC population; rather, in the specific study cohort selected, their predictive value for prognosis was limited.

The present study had some strengths. Notably, traditional Kaplan–Meier and Cox analyses deal with competing events only as censored observations, leading to an overestimation of the cumulative incidence rate and, sometimes, resulting in false positives and false negatives. In contrast, the use of a CRM could mitigate the estimation bias because the competing events and interesting outcomes are separately taken into consideration, and the effect of competing events on interesting outcomes is precluded in the CRM (49). Thus, the CRM approach could serve the role of non-GCSD for the endpoint of interest and to accurately estimate the cumulative incidence of interesting events. This was particularly important in a clinical study of GC since > 10% of the patients died because of causes other than GC in the present study. To the best of our knowledge, this is the first study with a large population to report the impact of LN status on the survival of patients with GC using the CRM approach. In addition, we focused on patients with LN-positive stage II GC undergoing resection and chemotherapy, such as a special population with a large yield of LN. An accurate staging system is needed to determine adequate treatment schedules and predict prognosis. Moreover, although TNM staging system is widely used, it has some inevitable deficiencies, especially when ELN is insufficient. We found that the LNR was superior to the ELN and pN in predicting the prognosis (including both OS and DSS) of patients with GC.

Our study also had some limitations. First, this was an analysis of the SEER database, which had the potential to include heterogeneous data with respect to diagnosis and treatment strategies over a long period. Second, since this was a retrospective study, the study design was limited, and potential bias could not be avoided. Third, data for some treatment and clinicopathological factors, such as LN dissection method, preoperative therapy, margin status, comorbidities, and chemotherapy regimen, were unavailable. These factors may also affect the prognosis of patients with GC. Finally, the sample size in our study was relatively small.

In conclusion, our results suggest that ELN, LNR, and pN are all significant predictors of OS for GC. However, the prognostic value of pN for DSS is likely to be influenced by death from other causes (competing events). LNR, in contrast, demonstrates stronger robustness in predicting DSS for GC than ELN and pN. Further prospective studies with larger sample sizes and more rigorous designs are required for a more accurate analysis of the role of the LNR in the prognosis of patients with GC and to identify the optimum LNR classification for application in clinical practice.

## Data Availability

The original contributions presented in the study are included in the article/**Supplementary Material**. Further inquiries can be directed to the corresponding author.
