# Supplementary figures and images for: Prognostic performance of examined lymph nodes, lymph node ratio, and positive lymph nodes in gastric cancer: a competing risk model study
Source: Front Endocrinol (Lausanne). 2025 Feb 21;16:1434999. doi: 10.3389/fendo.2025.1434999 (PMC11885136; doi:10.3389/fendo.2025.1434999)

Figure S1

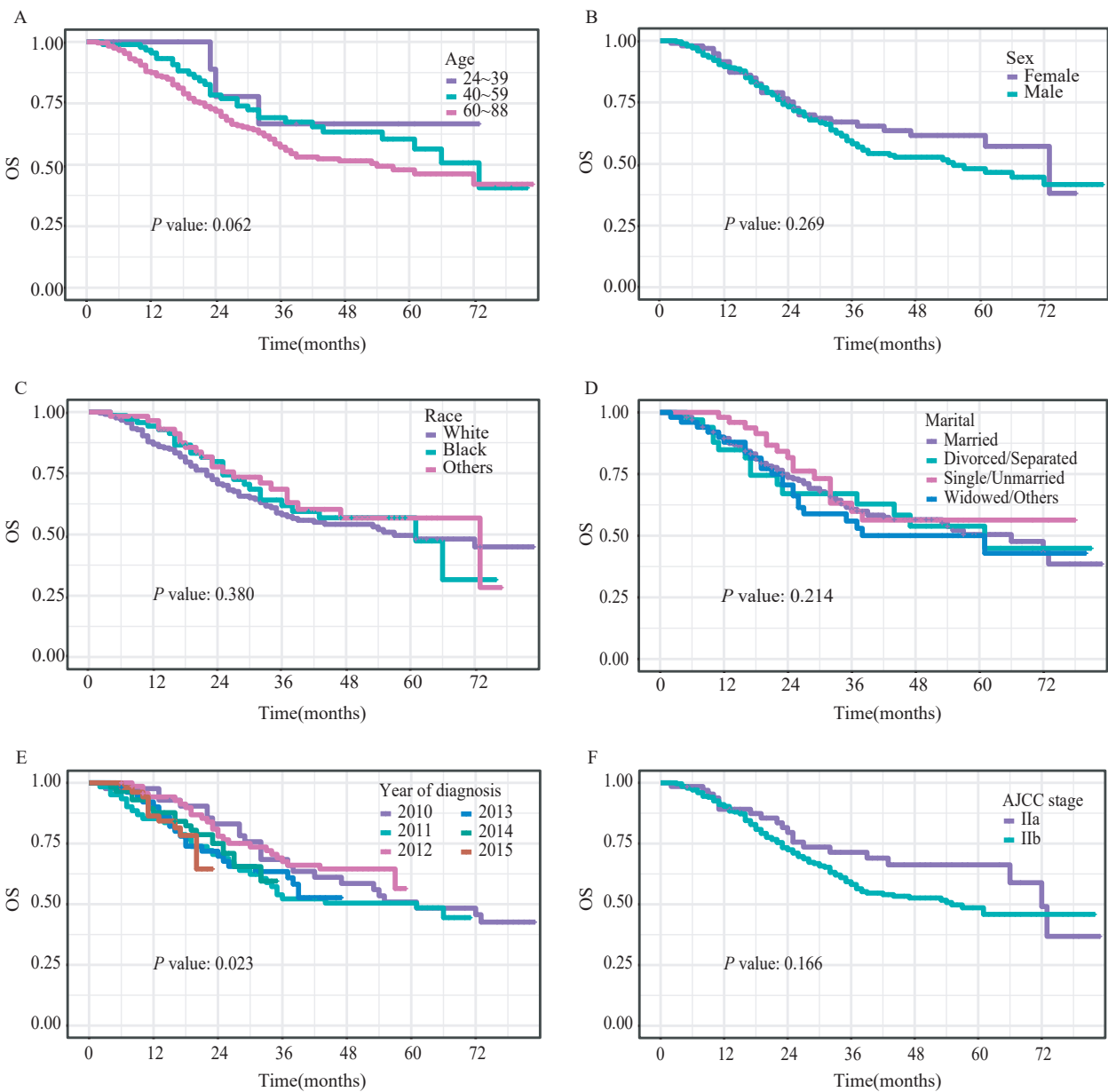

(continued on next page)

(continued)

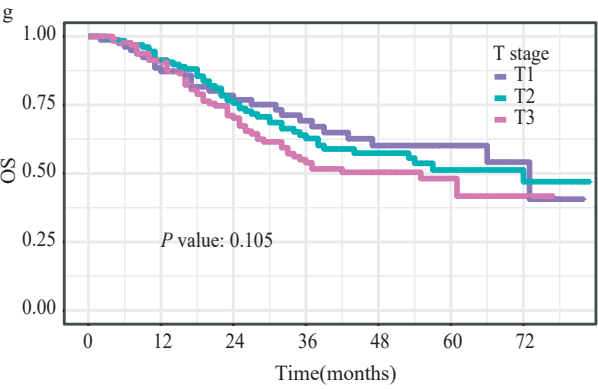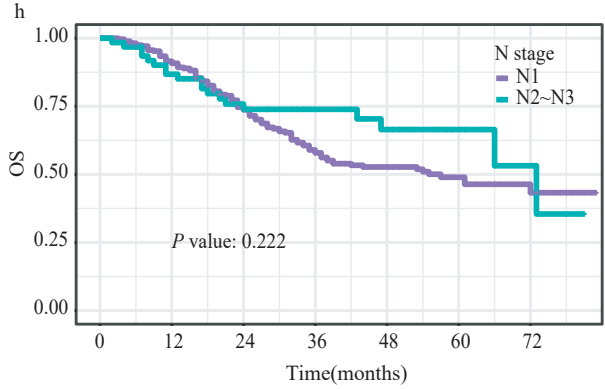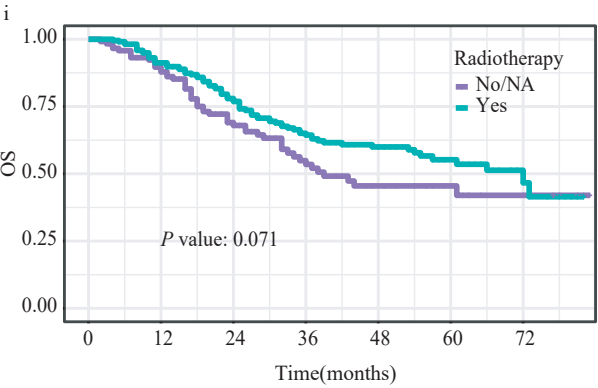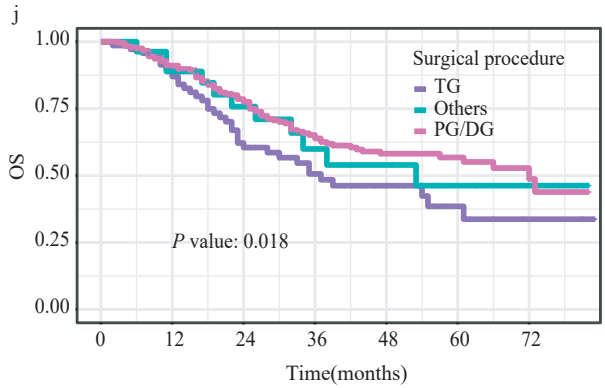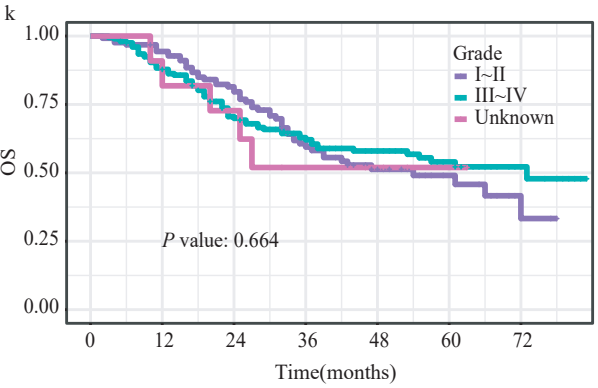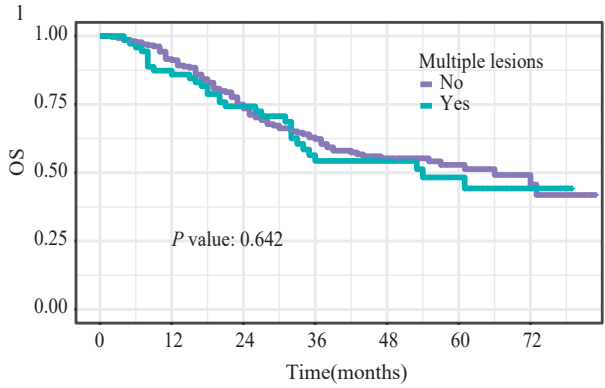

(continued on next page)

(continued)

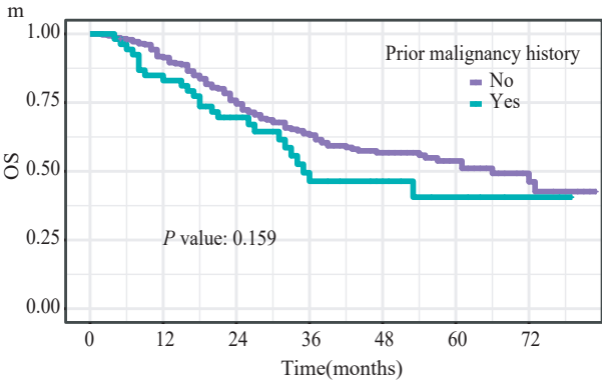

Supplement: Supplementary file 1 [file DataSheet1.pdf]

Figure S2

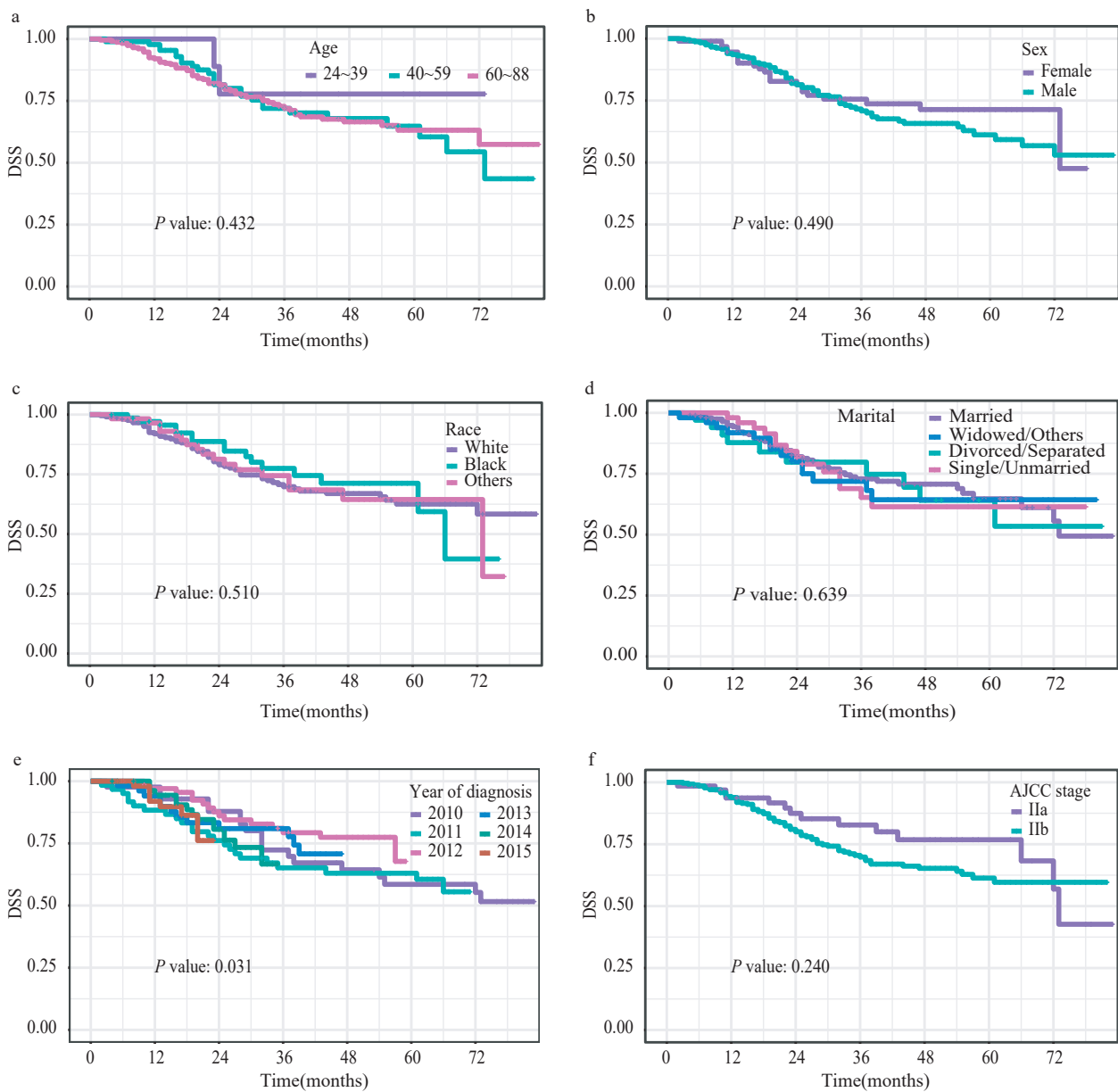

(continued on next page)

(continued)

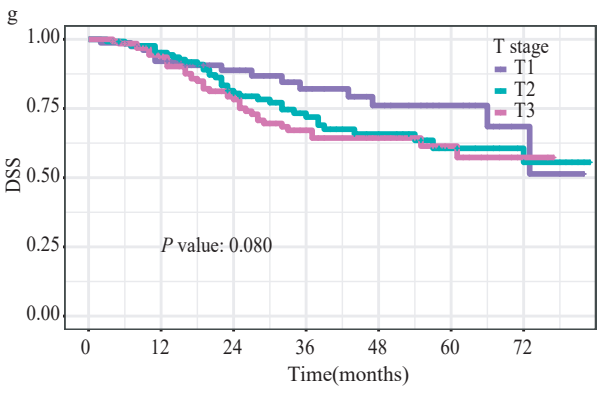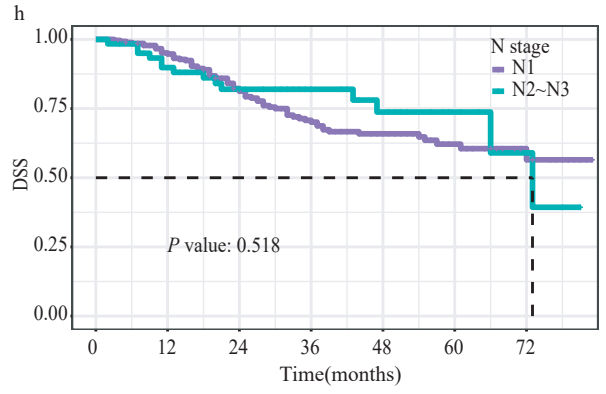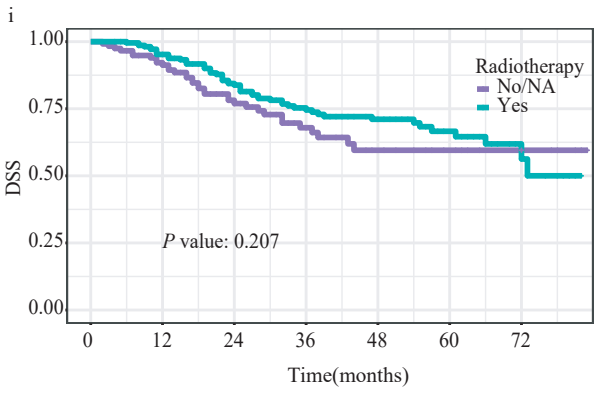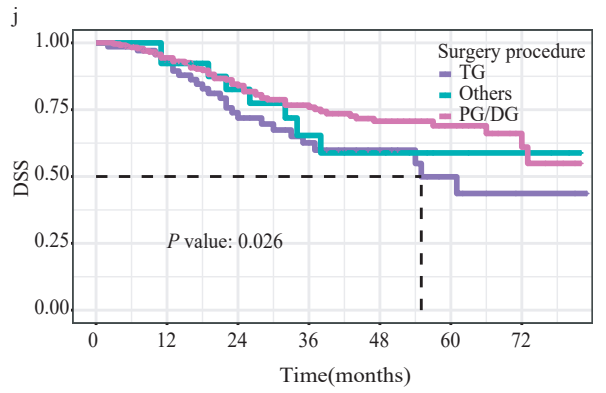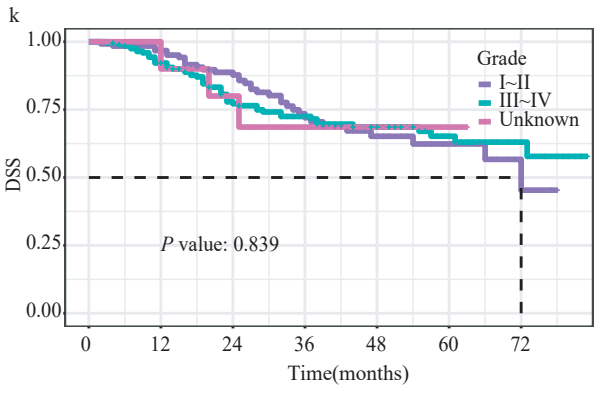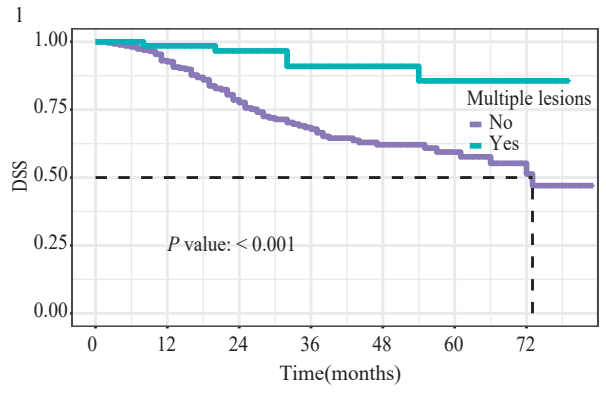

(continued on next page)

(continued)

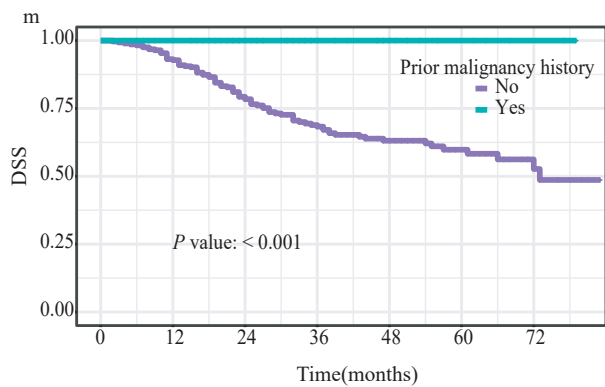

Supplement: Supplementary file 2 [file DataSheet2.pdf]

Figure S3

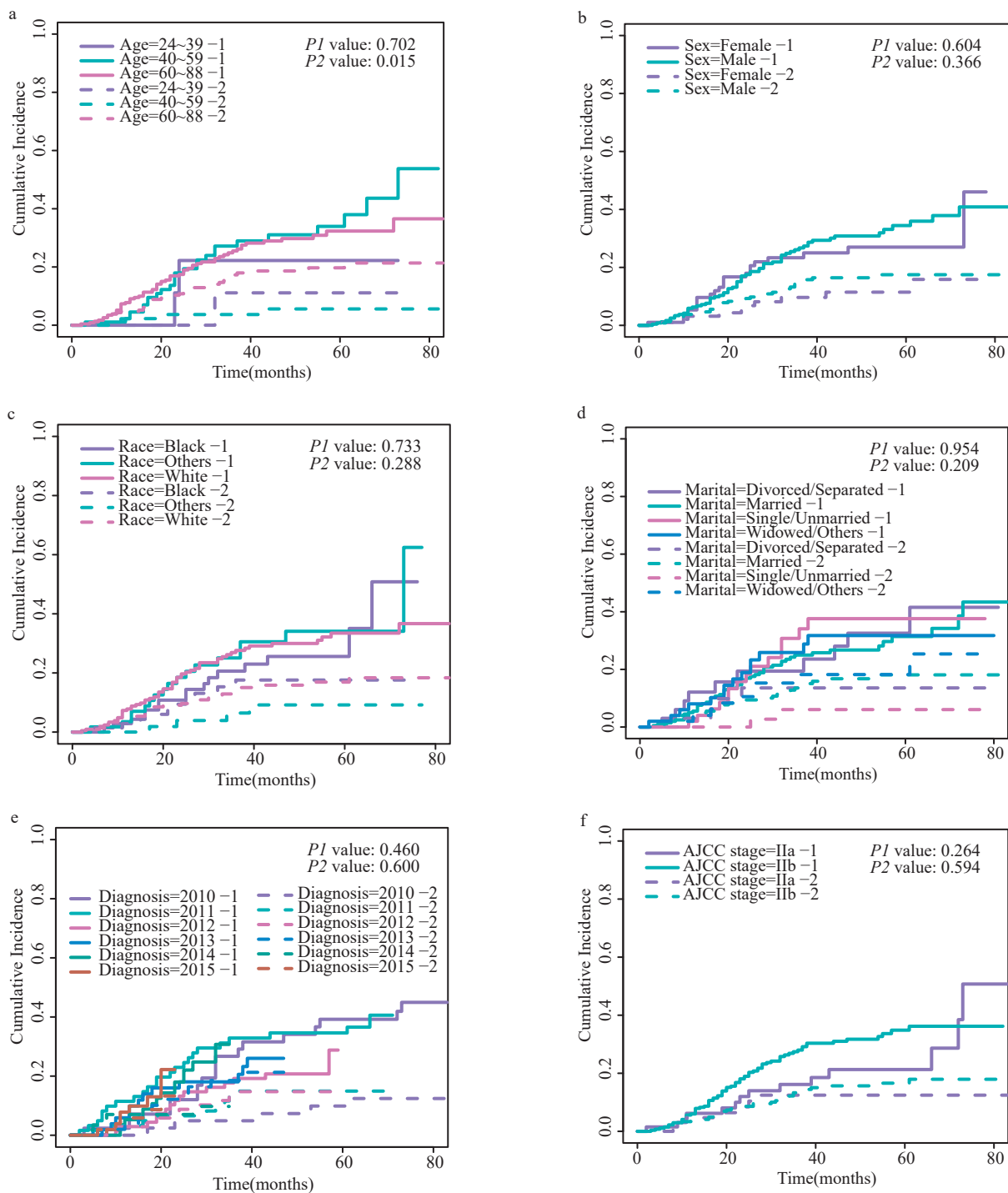

(continued on next page)

(continued)

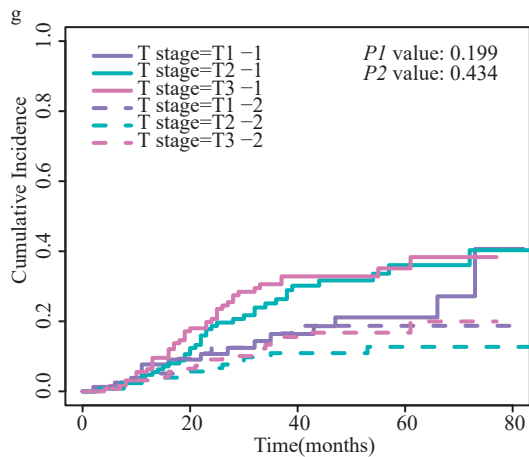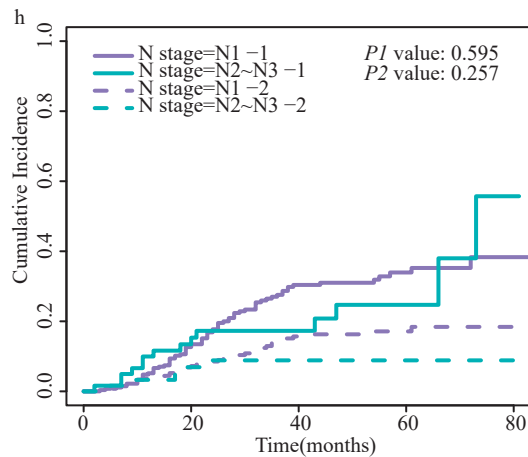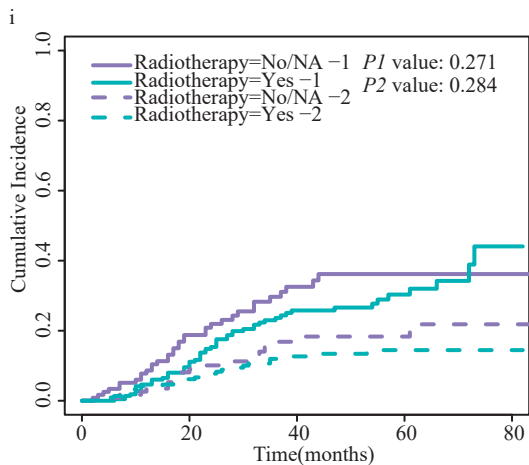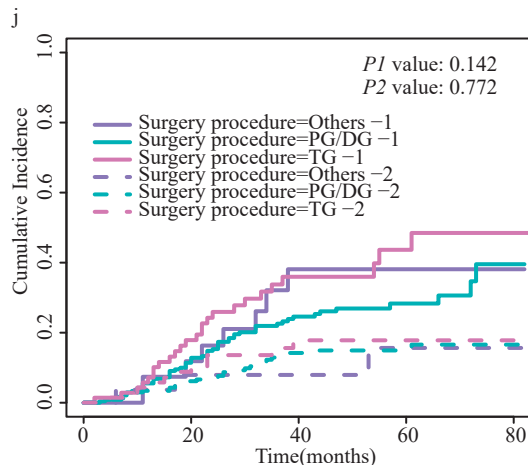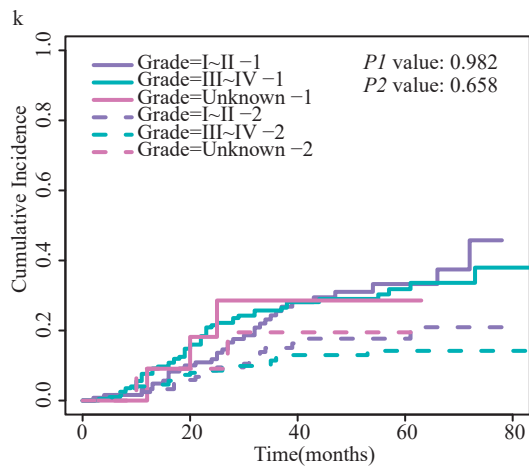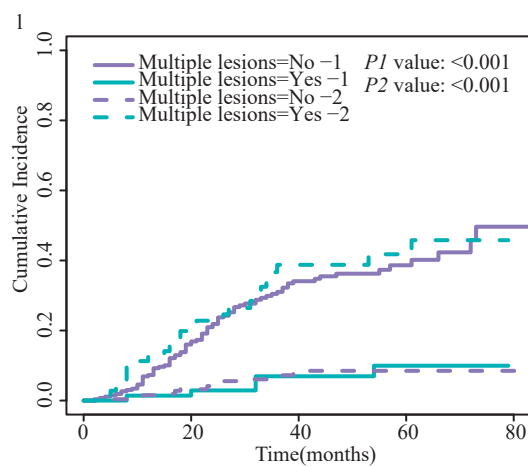

(continued on next page)

(continued)

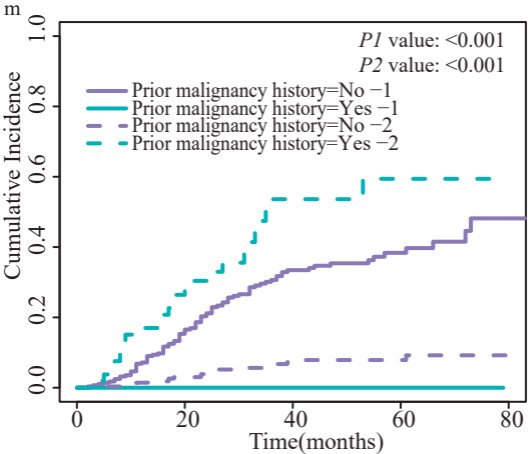

Supplement: Supplementary file 3 [file DataSheet3.pdf]
