# Supplementary material for: Prognostic performance of examined lymph nodes, lymph node ratio, and positive lymph nodes in gastric cancer: a competing risk model study
Source: Front Endocrinol (Lausanne). 2025 Feb 21;16:1434999. doi: 10.3389/fendo.2025.1434999 (PMC11885136; doi:10.3389/fendo.2025.1434999)
Supplement: Supplementary file 4 [file DataSheet4.pdf]

**Supplemental Figure Legends**

**Supplemental Fig. 1 Kaplan–Meier survival curves for OS stratified by clinicopathological characteristics.** (A) age, (B) sex, (C) race, (D) marital status at diagnosis, (E) year of diagnosis, (F) AJCC stage, (G) T stage, (H) N stage, (I) radiotherapy, (J) surgical procedure, (K) grade, (L) multiple lesions, (M) prior malignancy history. OS, overall survival; AJCC, American Joint Committee on Cancer; TG, total gastrectomy; PG/DG, proximal/distal gastrectomy; NA, not available

**Supplemental Fig. 2 Kaplan–Meier survival curves for DSS stratified by clinicopathological characteristics.** (A) age, (B) sex, (C) race, (D) marital status at diagnosis, (E) year of diagnosis, (F) AJCC stage, (G) T stage, (H) N stage, (I) radiotherapy, (J) surgical procedure, (K) grade, (L) multiple lesions, (M) prior malignancy history. DSS, disease-specific survival; AJCC, American Joint Committee on Cancer; TG, total gastrectomy; PG/DG, proximal/distal gastrectomy; NA, not available

**Supplemental Fig. 3 Cumulative incidence of GCSD and non-GCSD stratified by clinicopathological characteristics.** (A) age, (B) sex, (C) race, (D) marital status at diagnosis, (E) year of diagnosis, (F) AJCC stage, (G) T stage, (H) N stage, (I) radiotherapy, (J) surgical procedure, (K) grade, (L) multiple lesions, (M) prior malignancy history. The *P1* value represents the statistical result of comparing the cumulative incidence curves of GCSD between different groups using the Fine and Gray competing risks model. The *P2* value represents the comparison of cumulative incidence curves for non-GCSD between different groups. GCSD, gastric cancer-specific death; AJCC, American Joint Committee on Cancer; TG, total gastrectomy; PG/DG, proximal/distal gastrectomy; NA, not available
